# Electronic Nose for the Rapid Detection of Deoxynivalenol in Wheat Using Classification and Regression Trees

**DOI:** 10.3390/toxins14090617

**Published:** 2022-09-03

**Authors:** Marco Camardo Leggieri, Marco Mazzoni, Terenzio Bertuzzi, Maurizio Moschini, Aldo Prandini, Paola Battilani

**Affiliations:** 1 Department of Sustainable Crop Production, Università Cattolica del Sacro Cuore, Via E. Parmense 84, 29122 Piacenza, Italy; 2 Department of Livestock Population Genomics, University of Hohenheim, Garbenstraβe 17, 70599 Stuttgart, Germany; 3 Department of Animal Science, Food, and Nutrition, Università Cattolica del Sacro Cuore, Via E. Parmense 84, 29122 Piacenza, Italy

**Keywords:** e-nose, *Fusarium graminearum*, mycotoxin, machine learning, small grains, metal oxide sensors, DON

## Abstract

Mycotoxin represents a significant concern for the safety of food and feed products, and wheat represents one of the most susceptible crops. To manage this issue, fast, reliable, and low-cost test methods are needed for regulated mycotoxins. This study aimed to assess the potential use of the electronic nose for the early identification of wheat samples contaminated with deoxynivalenol (DON) above a fixed threshold. A total of 214 wheat samples were collected from commercial fields in northern Italy during the periods 2014–2015 and 2017–2018 and analyzed for DON contamination with a conventional method (GC-MS) and using a portable e-nose “AIR PEN 3” (Airsense Analytics GmbH, Schwerin, Germany), equipped with 10 metal oxide sensors for different categories of volatile substances. The Machine Learning approach “Classification and regression trees” (CART) was used to categorize samples according to four DON contamination thresholds (1750, 1250, 750, and 500 μg/kg). Overall, this process yielded an accuracy of >83% (correct prediction of DON levels in wheat samples). These findings suggest that the e-nose combined with CART can be an effective quick method to distinguish between compliant and DON-contaminated wheat lots. Further validation including more samples above the legal limits is desirable before concluding the validity of the method.

## 1. Introduction

Trichothecenes and zearalenone (ZEN) may occur in cereal grains with *Fusarium* head blight (FHB) associated with a complex of *Fusarium* species [1,2]. Trichothecenes are classified into two groups: type A and type B. Type B trichothecenes, produced mainly by *Fusarium graminearum* and *F. culmorum*, are prevalent and include deoxynivalenol (DON) as the predominant compound, commonly co-occurring with 3- and 15-acetyl-deoxynivalenol (3-Ac-DON and 15-Ac-DON) and nivalenol (NIV). DON is the most widespread mycotoxin in wheat, with durum wheat more contaminated than soft wheat [3]; this is confirmed in Italian wheat grain production. Type A trichothecenes include T-2 and HT-2 toxins produced by *F. langsethiae* and *F. sporotrichioides*; type A are more toxic than type B trichothecenes, but type A commonly occur in lower amounts. Due to their stability, even during food processing, trichothecenes and ZEN are implicated in human and animal health issues [4]. The European Commission [5] established maximum residue levels (MRL) for the presence of DON and ZEN in unprocessed cereals for human consumption: 1250 and 1750 μg/kg for DON in soft and durum wheat, respectively, and 100 μg/kg for ZEN in all cereals other than maize. In addition, MRL for T-2 and HT-2 toxins are under discussion.

Soft and durum wheat are essential crops worldwide; regarding Italy, soft wheat is grown mainly in the north (about 75%), with durum wheat prevalent in central and southern areas [6]. In the past decade, north Italy has increased its contribution to durum wheat production (about 13% of the Italian output and 18% of the wheat produced in northern Italy): the Emilia-Romagna region contributed about 30% and 9% to soft and durum Italian wheat production, respectively [6]. Due to the regulation in force, stakeholders of the wheat grain chain must check the lots for compliance with the law regarding DON contamination. This operation is crucial when grain is delivered from farmers to storehouses or processors because they need to know the contamination level; nevertheless, this is a bottleneck because those who are delivering want to discharge rapidly, and time elapsed from arrival to destination and discharge is time lost. Therefore, rapid methods that can be applied at grain delivery are a necessity for the acquiring and delivering actors in this process.

The presence of DON in cereals and cereal-based products is mainly detected by chromatographic methods such as high-performance liquid chromatography (HPLC or UHPLC) coupled with ultraviolet (UV), diode array (DAD), or mass spectrometry (MS) detectors, and gas-chromatography (GC) coupled with electronic capture (ECD) or MS detectors [7,8]. These methods gave reliable results, but they need time, expertise, proper machines, and they are expensive. In regard to rapid analytical approaches that provide qualitative or semi-quantitative results, the electronic nose (e-nose) has attracted great interest regarding food and feed quality control [9,10,11], and it has been used in various commercial agriculture-related industries [12]. E-nose, through its sensors delivers a smell-print of samples, which can be learnt based on a pattern recognition approach [13,14]. E-nose could be a valuable method for screening grain lots at delivery due to its rapidity and low cost in classifying food/feed matrices with various chemical “fingerprints”. Lippolis et al. [15] applied e-nose to naturally contaminated durum, wheat either on whole or ground kernels, but correct attributions regarding contamination, obtained with Discriminant Function Analysis, were higher than 80% only for ground grain. 

The e-nose coupled with machine learning (ML) was recently proposed by Camardo Leggieri et al. [11] to classify maize samples by discriminating between non-contaminated and contaminated samples; the reference thresholds used were based on the EU legislation for aflatoxin B1 and fumonisins. The advantage in coupling ML to detection methods was also underlined for the detection of wheat quality in artificially inoculated grains [16] or for other agricultural aims, among others, early detection of moldy apples [17] or aphid infestation in wheat [18].

Screening undesired compounds at grain delivery makes it possible to properly address sorting operations aimed at reducing the occurrence of contaminated grains and preventing contaminated lots from entering the storage/processing step. Interesting approaches have been recently developed for sorting purposes, based on single kernel analysis. The most potent tools described are based on NIR-spectrometry and Hyperspectral Imaging (HSI), and very good performances were reported both for fungi and mycotoxin detection [13,19]. This technique can be used as a mitigation strategy for the removal of highly contaminated grains from cereal batches. Therefore, e-nose, or other rapid methods, such as infrared spectrometry, supported by a robust data analysis, can be applied to identify highly contaminated lots [20] to be addressed to single kernel selection and analyzed later with more precise methods to confirm the compliance of the materials with the legal limits. In reality, this is a great advantage, reducing the time delay and high cost of more tedious laboratory analyses, which is only required upon testing positive during on-site screening [20].

This work aimed to extend the usage of e-nose as a rapid method for evaluating DON contamination in wheat lots, following an approach comparable to Camardo Leggieri et al. [11]. The number of fields monitored was lower than in the maize study, which imposed a different data analysis, but four different contamination thresholds were introduced.

## 2. Results

### 2.1. Field Sampling and Deoxynivalenol Contamination

A total of 214 wheat samples contributed to the DON dataset, with 50–57 samples collected yearly (Table 1). Samples were collected in four different areas in the Emilia-Romagna region (northern Italy), belonging to the province of Bologna (20%), Ferrara (54%), Modena (9%), and Ravenna (17%). The incidence of samples above the limit of quantification (LOQ) and mean contamination value varied between years. Both the highest and the lowest (>LOQ) DON content were detected in 2017, 14,829 μg/kg and 20 μg/kg, respectively; samples with DON content < LOQ were found in 2014 and 2015. The yearly average DON contamination was lower than 500 μg/kg for 2014 and 2015 but higher than 700 μg/kg for 2017 and 2018. 

### 2.2. Data Analysis 

Four different thresholds were considered, 1750, 1250, 750, and 500 μg/kg, and samples were clustered in four distinct groups. In the original data set, samples with DON contamination above the clustering threshold of 1750, 1250, 750, and 500 μg/kg were 8%, 9%, 16%, and 23%, respectively. Two further data sets were generated from the original one, training and blind data sets, keeping the same proportion of positive and negative samples. Detailed information on the distribution of DON contaminated and non-contaminated wheat samples, based on different thresholds, are listed in Table 2.

The e-nose sensors measures were used as input features and put in relation to mycotoxin contamination following the same approach of Camardo Leggieri et al. [11], without satisfactory results. Therefore, the Classification and Regression Tree (CART) was implemented.

**Figure 1:**
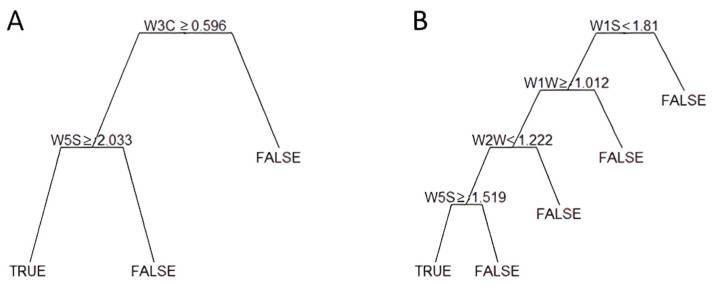
Example of tree classifiers for two selected thresholds of mycotoxin contamination: (**A**) 1750 μg/kg, (**B**) 500 μg/kg. Starting from the top, only one sensor is considered (i.e., W3C in (**A**)). If the condition is matched, the second sensor is assumed; otherwise, the negative class is assigned (FALSE in the figure), and another sensor is considered.

Figure 1 shows the CART output for the extreme thresholds (1750 and 500 μg/kg). The CART used data generated by two sensor arrays to classify samples above the threshold of 1750 μg/kg (Figure 1A); in particular, W3C and W5S sensors accounting for ammonia (used as a sensor for aromatic compound) and broad range sensitivity (nitrogen oxides and ozone), respectively. The same sensors were used for the threshold 1250 μg/kg.

The CART utilized four sensors (Figure 1B) to split samples above the 500 μg/kg threshold. In addition to the W5S mentioned above, in both cases, accounting for the last step of the CART, sensors W2W, W1W, and W1S, specific for aromatic compounds (organic sulfur compounds), sulfur compounds, and methane, were considered instead of W3C. 

The coincidence matrix of DON (Table 3) for the threshold 1750 μg/kg showed that CART correctly classified about 92% of samples. The correct classifications slightly decreased with lower thresholds; in particular, 90%, 90%, and 84% of correct classifications for 1250, 750, and 500 μg/kg, respectively. Incorrect classifications included approximately 5%, 5%, 2% and 2% underestimates and 3%, 5%, 8%, 14% overestimates for 1750, 1250 and 750 and 500 μg/kg, respectively (Table 3).

### 2.3. Cross-Validation with Training and Blind Dataset

Regarding the assessment capability of the e-nose, for each threshold, five-cross validation methods were performed using the Training Dataset (Table 4). The accuracy (ACC) was 0.91–0.92 for the higher classification threshold (1750, 1250, and 700 μg/kg) and 0.85 for the threshold = 500 μg/kg. Regarding the Blind Dataset, the ACC was higher than 0.81 for all the considered thresholds and consistently lower than the ACC of the Training Dataset. The index was higher in the Training Dataset for all the thresholds considered regarding the Blind Dataset. The e-nose sensitivity (or TPR) was consistently higher for the Training Dataset compared to the Blind Dataset for all the four tested thresholds, while the specificity (or TNR) was ≥0.94 for all the thresholds regardless of the dataset. Finally, higher precision (or PPV) was consistently observed for the Training Dataset with respect to the Blind Dataset at all the tested classification thresholds, with the highest PPV for the thresholds set at 750 and 500 μg/kg.

## 3. Discussion

Developing reliable and rapid methods for mycotoxin detection is a priority [21]; mycotoxins are food contaminants, and legal limits for the maximum content allowed in different matrices have been set in most countries worldwide. The e-nose has been identified in many studies as an effective tool for rapidly screening food substances [15,22]. Recently, the e-nose, in combination with ML techniques, has been very successful when applied to maize naturally contaminated with mycotoxin [11]. The current research used the same approach to evaluate DON contamination in wheat. In total. 214 wheat samples were analyzed with the e-nose; this dataset did not allow for the application of the same ML approach previously used for maize. However, good results were obtained with the ML-CART, which was applied to separate contaminated and non-contaminated samples. 

Four different DON thresholds were set in this study to share positive (above the threshold) and negative samples. Two thresholds, 1750 and 1250 μg/kg, match the legal limits set by the European Union [5] for durum and soft wheat, respectively. Two additional values, 750 and 500 μg/kg, were included to pursue a more balanced distribution of samples above and below the threshold, and to assess the potentiality of the e-nose to discriminate samples for more restrictive contamination levels. The number of positive samples was approximately 8–9% for the highest thresholds and 16 and 23% for 750 and 500 μg/kg, respectively. The e-nose is known to produce qualitative output responses for mycotoxins, the presence or absence of mycotoxins at fixed thresholds; in this study, the use of four different thresholds makes this attempt semi-quantitative. 

The highest accuracy in sample attribution (0.88–0.92) was obtained for the cut-off values ≥ 1250 μg/kg, with 87–89% correct predictions, the same correct classification reported in the previous study [10]. These thresholds are the official ones; therefore, the result is quite important, and its validity is robust because the tested samples were collected in four growing seasons and from different locations in north Italy. Rapid analytical methods, including e-nose, gave variable results with samples derived from different years or geographic areas; therefore, the high accuracy obtained with this dataset allows us to also speculate on the validity of the approach with other datasets [23,24].

Unfortunately, increased incorrect predictions were found with the lower threshold (μ ≤ 750 μg/kg), and this effect may be due to the lower sensitivity of the instrument at a lower concentration of DON; this hypothesis is supported by the number of sensors used by the CART to classify samples according to the thresholds. Only two sensors have been used for the threshold of 1750 μg/kg and four for 500 μg/kg. In particular, the most critical sensors used by the e-nose, selected through the CART, were W3C and W5S for the threshold of 1750 μg/kg, which can detect ammonia and broad range molecules, respectively. In past studies, only acetates and γ-caprolactone were associated as volatile compounds with DON [15,25], but due to the application of the HS-SPME/GC-MS technique. However, the ability of an e-nose to detect volatile compounds associated with mycotoxins contamination depends on the sensor’s sensitivity to the various compounds. This has recently been tested by Machungo et al. [26]. They considered three portable e-nose devices to compare the performance of metal oxide sensors (MOS) and conducted polymer noses to detect aflatoxins in artificially contaminated maize; MOS was the most effective for mycotoxin detection. In our study, the e-nose was a portable device equipped with 10 MOS sensors. Combined with the ML approach, it gave good results in categorizing the occurrence of aflatoxin and fumonisin in maize [11], as previously reported.

Only W5S sensor, used for sample classification, characterized by the broadest range sensitivity among the sensors used, was common among data sets generated with different thresholds. This is not surprising; in fact, e-nose sensors give a qualitative answer, as they respond to families of volatile compounds and are not specific. Increasing the number of samples, some additional substances may be considered only because they are present in some samples, and this could involve the selection of an additional sensor or the leakage of other sensors. In addition, volatiles result from the interaction between plants and fungi, and they are tuned by the severity of the fungal attack and the susceptibility of the host crop [27,28]. Therefore, changing the threshold, the contribution of samples changes, as do the volatiles perceived by the sensors.

Other researchers have also tested and discussed the potentialities of the e-nose. Campagnoli et al. [29] performed a discriminant analysis using a data set of 30 wheat samples, 22 above the LOD (50 μg/kg), and obtained an accuracy of 100%. However, the number of samples considered in the study was small, and the reliability of the e-nose technology based on such a dataset is questionable. A further e-nose application, including 122 durum wheat samples, was managed in 2011 by Campagnoli et al. [30] to predict DON content, using the principal component analysis (PCA) to assess three different contamination clusters as non-contaminated and contaminated below/above the EU limit of 1750 μg/kg; a 3.3% error rate was obtained. As expected, the rate of correct predictions slightly decreased, increasing the number of samples analyzed from 30 [29] to 122 [30], confirming the importance of a reasonable number of samples to gain reliable predictions. Another example on a similar food/feed matrix (wheat bran) was performed in a broader dataset with 470 samples naturally contaminated by DON and was used to test the e-nose potentiality at the cut-off threshold of 400 μg/kg; 89% of correct classification using discriminant analysis (DA) was reached, utilizing samples with DON ≥ 400 μg/kg [10]. Therefore, irrespective of the number of samples used, e-nose gave good results.

This is the first study using wheat samples naturally contaminated by DON collected in four different years and combining an e-nose with an ML-CART analysis. The accuracy obtained, regardless of the sensors that contributed significantly, offer an insight into the potential application of e-nose as a diagnostic technique for untargeted preliminary screening of wheat samples, based on different thresholds of contamination, to reduce the number of samples requiring more expensive and time-consuming chemical analysis. The weak point that should be underlined is the limited number of samples contaminated above the legal limits occurring in the data set: only 8–9, a maximum of 23%, were clustered positive with 500 μg/kg as a threshold. 

## 4. Conclusions

As repeatedly mentioned in the text, to conclude, e-nose is a screening method and cannot be used as an alternative to more reliable and precise methods based on HPLC or mass spectrometry. Nevertheless, practical problems to be solved by crop value chain operators should be considered. The rapid acquirement of information regarding the compliance of cereal lots with the regulation in force is crucial, even if confirmations are requested. The data set used in this study is comprehensive but due to the source of variation mentioned, to conclude on the validity of the method, further validation with a more balanced data set is desirable. The acquirement of further data, both for soft and durum wheat, would be quite useful to confirm the proposed approach’s performance and possibly improve its accuracy with a tailored data analysis for the two *Triticum* species. 

## 5. Materials and Methods

### 5.1. Field Sampling and Laboratory Sample Preparation

#### 5.1.1. Samples

Over the harvest years 2014, 2015, 2017, and 2018, 214 wheat samples were collected in fields distributed in the Emilia-Romagna region (Northern Italy) at full ripening, according to EC Regulation 401/2006 [31]. For mycotoxin quantification, a final grain sample of 7–10 kg for each field was delivered to the laboratory. A lab sample of 200 g was randomly taken from each field for e-nose analysis; the remaining samples were milled and homogenized with a cyclone hammer mill (1 mm sieve, Pulverisette, Fritsch GmbH, Idar-Oberstein, Germany). After milling and homogenization, an aliquot of 2 kg was stored at –20 °C until the time of mycotoxin analysis.

#### 5.1.2. e-Nose Analysis

The e-nose used in this study was a portable “AIR PEN 3” (Airsense Analytics GmbH, Schwerin, Germany) equipped with 10 metal oxide sensors (MOS) for different categories of detectable volatile substances (Table 5). The e-nose was equipped with pattern recognition software for data recording and processing (WinMuster, v. 1.6.2.13). As Camardo Leggieri et al. [11] reported, e-nose parameters were set. Briefly, a calibration procedure was applied each day of e-nose use and reference sensor response was recorded (G_0_). The aliquot of 100 g of wheat grain was placed into 250 mL round bottom flasks and left to stand for one hour at 25 °C to allow a build-up of volatiles in the headspace before e-nose sampling. The analysis was managed for 60 s, registering each second the signals for each sensor (G), reported as conductance ratio G/G0. E-nose sensor outputs were used in data analysis and associated with DON contamination, according to Camardo Leggieri et al. [11].

### 5.2. Mycotoxin Analysis

Analyses and standard preparations were performed according to methods reported by Bertuzzi et al. [23]; briefly, DON was extracted using a mixture CH_3_CN:H_2_O = 84:16 and a volume of 6 mL was eluted through a SPE column (PuriTox Trichothecene, R-Biopharm, Darmstadt, Germany). Determination was carried out by GC-MS after derivatisation (limit of detection, LOD: 3 μg/kg and limit of quantification, LOQ: 10 μg/kg). 

### 5.3. Data Analysis

Deoxynivalenol (DON) concentration was used to cluster samples in contaminated and not contaminated. Four different thresholds were considered, 1750, 1250, 750, and 500 μg/kg, giving four distinct sample clusters. The thresholds 1750 and 1250 μg/kg correspond to the maximum level of DON in unprocessed cereals, particularly for durum and soft wheat, respectively (EC 2006 [5], 2007 [32]). For each sample clustering, Class 1 was assigned to all the samples exceeding the fixed threshold and class 0 to all samples equal to or below it. 

The relationship between DON occurrence and e-nose data was analyzed following the same approach used in Camardo Leggieri et al. [11]. Briefly, three methods were used: 1. Artificial Neural Network (ANN); 2. Discriminant Analysis (DA); and 3. Logistic Regression (LR) analysis. However, none of the previously cited methods reached satisfactory results, mainly due to the high unbalance (many samples with class 0 vs. very few samples with class 1) of the dataset considered.

Therefore, the machine learning (ML) algorithm Classification and Regression Tree (CART) was implemented using R (v4.0.3) and the package “Caret” [33].

#### 5.3.1. Classification and Regression Trees (CART)

The CART is an ML approach that divides the dataset into smaller and smaller groups through repeated binary splits. At each step, only one e-nose sensor is considered. The algorithm then uses the value of the sensors to divide the space into smaller regions until a region is assigned to a class label 0 or 1, non-contaminated (0, negative) or contaminated (1, positive) in this study. An impurity function is computed at each step to measure how good a split is. The algorithm is defined based on an impurity function, which determines how good the classification is at each partition. The algorithm keeps the division that minimizes the impurity function at each step. This is achieved when many samples belong to a class in that part of the space, and a few belong to the other. Finally, a stop criterion is introduced to avoid overfitting. During the training process “*Caret*” requires some specific parameters to compute the model. The metric “accuracy” has been specified to select the best one. Cross-validation was implemented as a 5-fold. Then, a complexity factor (cp) used to compute the optimal tree was used. For the purpose of this study, a cp of 0.001 was used.

The CART is a supervised approach, like many other ML algorithms, and it requires a training phase with labeled samples (training data set) to create a suitable model for further classification or regression purposes. The CART has advantages which were helpful during the entire classification process. It is quick to implement and requires very low data pre-processing; the output of the sensors can be used as they are, while other ML algorithms require different pre-processing methods such as outliers filtering, normalization, or standardization to achieve consistent results. Furthermore, the CART output is easily represented and understood, unlike, for example, ANNs, which are black boxes, and it is almost impossible to understand where the error occurred during a misclassification [34]. Therefore, using this approach, e-nose sensor outputs were related to DON contamination and run four times, one for each threshold used for clustering.

#### 5.3.2. Model Evaluation

The four models obtained with the CART approach, one for each threshold used, were evaluated as described in Camardo Leggieri et al. [11]. Briefly, two datasets were randomly generated, maintaining the proportion of contaminated versus non-contaminated samples using the built-in function “*createDataPartition*” of the R package “*Caret*” [33]. 

This is essential to balance datasets and obtain reliable results. These two datasets were named Training and Blind Datasets; the Blind Dataset accounted for ~30% of the whole dataset. Feature importance was computed during the training; if a variable is not significant for the classification, it is removed and thus not considered [34]. A 5-fold Cross-validation (CV) was performed on the Training Dataset, and then the quality of the model was computed on the Blind Dataset. Several statistical scores were computed both for the Training Dataset and Blind Dataset for the evaluation of the models:

Accuracy (*ACC*):

(1) $$\begin{array}{c} ACC=\frac{TP+TN}{TP+TN+FP+FN} \end{array}$$

True Positive and True Negative rates (*TPR*, *TNR*):

(2) $$\begin{array}{c} TNR=\frac{TN}{TN+FP} \end{array}$$

Positive Predictive Value (*PPV*):

(3) $$\begin{array}{c} PPV=\frac{TP}{TP+FP} \end{array}$$

Balanced Accuracy (*BA*):

(4) $$\begin{array}{c} BA=\frac{\frac{TP}{TP+FN}+\frac{TN}{TN+FP}}{2} \end{array}$$

## Figures and Tables

**Table 1:** Descriptive statistics of deoxynivalenol (DON) content (μg/kg) in wheat grain samples collected in Emilia Romagna in 2014–2018 (except for 2016).

Year	N #	Mean	StDev	Minimum	Maximum
2014	52	98	126.4	<LOQ *	615
2015	55	205	233.9	<LOQ **	1171
2017	57	1069	2208.4	20	14,829
2018	50	1147	2217.9	59	10,898

(# Number of wheat samples; * LOD: 3 μg/kg; ** LOQ: 10 μg/kg).

**Table 2:** Distribution of the number of contaminated (positive) and non-contaminated (negative) wheat samples based on different thresholds of deoxynivalenol (DON) content (1750 μg/kg, 1250 μg/kg, 750 μg/kg, and 500 μg/kg), used for Training and Blind Dataset.

Threshold (μg/kg)	Original Dataset		Training Dataset		Blind Dataset	
	Positive	Negative	Positive	Negative	Positive	Negative
1750	18	196	13	138	5	58
1250	20	194	14	136	6	58
750	34	180	24	126	10	54
500	49	165	35	116	14	49

**Table 3:** Coincidence matrices computed from Blind Dataset results for the predicted and observed values of deoxynivalenol (DON; μg/kg): DON contamination data were shared based on four different thresholds (1750 μg/kg, 1250 μg/kg, 750 μg/kg and 500 μg/kg). Predictions against observed results were reported as percentages. Grey cells contribute to correct predictions for each threshold fixed; the white cell on the right indicates underestimates (observed positive and predicted negative), the white cell on the left indicates overestimates (observed negative and predicted positive).

Thresholds (μg/kg)	Observed	Predicted	
		Positive (%)	Negative (%)
1750	Positive	3	5
	Negative	3	89
1250	Positive	3	5
	Negative	5	87
750	Positive	5	2
	Negative	8	85
500	Positive	6	2
	Negative	14	78

**Table 4:** Summary of results for deoxynivalenol (DON) prediction in wheat samples. Results of the cross validation with the Training Dataset (TD) and Blind Dataset (BD) of each tested threshold (1750 μg/kg, 1250 μg/kg, 750 μg/kg, and 500 μg/kg) were reported.

Threshold (μg/kg)	1750		1250		750		500	
Data Set	TD	BD	TD	BD	TD	BD	TD	BD
Cross validation method								
* ACC	0.92	0.89	0.91	0.88	0.91	0.81	0.85	0.83
TPR	0.31	0.50	0.29	0.40	0.54	0.37	0.40	0.36
TNR	0.98	0.97	0.98	0.97	0.98	0.94	0.99	0.96
PPV	0.57	0.40	0.57	0.40	0.87	0.75	0.93	0.71
BA	0.64	0.72	0.63	0.67	0.76	0.567	0.70	0.66

* ACC, Accuracy; TPR, True Positive Rate or Sensitivity; TNR, True Negative Rate or Specificity; PPV, Positive Predictive Value or Precision; BA, Balanced Accuracy.

**Table 5:** Sensitivity and selectivity of the sensor array in the portable electronic nose device (PEN 3 Portable Electronic Nose, Airsense Analytics GmbH, Schwerin, Germany).

Number in Array	Sensor	General Description	Reference
1	W1C aromatic	Aromatic compounds	Toluene, 10 ppm
2	W5S broad range	Broad range sensitivity, react on nitrogen oxides and ozone, very sensitive with negative signal	NO_2_, 1 ppm
3	W3C aromatic	Ammonia, used as sensor for aromatic compounds	Benzene, 10 ppm
4	W6S hydrogen	Mainly hydrogen, selectively (breath gases)	H_2_, 100 ppb
5	W5C aromatic-aliphatic	Alkanes, aromatic compounds, less polar compounds	Propane, 1 ppm
6	W1S broad methane	Sensitive to methane (environment) ca. 10 ppm, broad range, similar to W2S	CH_4_, 100 ppm
7	W1W sulphur organic	Reacts on sulphur compounds (H2S 0,1 ppm), otherwise sensitive to many terpenes and sulphur organic compounds, which are important for smell (limonene, pyrazine)	H_2_S, 1 ppm
8	W2S broad alcohol	Detects alcohol’s, partially aromatic compounds, broad range	CO, 100 ppm
9	W2W sulphur-chlorine	Aromatic compounds, sulfur organic compounds	H_2_S, 1 ppm
10	W3S methane-aliphatic	Reacts on high concentrations > 100 ppm sometimes very selective (methane)	CH_4_, 100 ppm
